# Book Reviews

**Published:** 1833-08

**Authors:** 


### CRITICAL ANALYSES.

Quæ laudanda forent, et quæ culpanda, vicissim,
Illa prius, cretâ; mox hæc, carbone, *notamus*.—Persius.

Asthma. [Dr. Copland's *Dictionary of Practical Medicine.*]

Dr. Copland has given, in the space of a few pages, so much valuable information upon the subject of asthma, that we feel persuaded we cannot select a more profitable topic for the notice of our practical readers. He defines asthma to consist in great difficulty of breathing, recurring in paroxysms, accompanied with a wheezing sound, sense of constriction in the thorax, anxiety, and a difficult cough, terminating in mucous expectoration. There are few diseases the nature of which has been a subject of greater doubt and difference of opinion than asthma. In proof of this diversity of opinion, Dr. C. remarks that, until the writings of Floyer, Willis, Hoffmann, Alberti, and Juncker, directed particular attention to its pathology, it was generally confounded with dyspnœa, being usually denominated intermittent or remittent dyspnœa. By these writers, and more recently by Sauvages, Cullen, Pinel, and Georget, asthma was considered as essentially nervous in its nature; and the lesions found upon the dissection of fatal cases viewed as its consequences, and not as its causes. More recently, and even at the present day, among many, it has been considered as a symptom of organic change of either the heart, large blood-vessels, or of the lungs, air-tubes, &c. But this doctrine, in Dr. C.'s opinion, although generally accurate in respect of dyspnœa, is quite erroneous as applied to asthma.

"*Pathology of Asthma.* The dependence of dyspnœa, not only upon organic lesions of the organs seated within the chest, but upon the form of the thorax, upon diseases of adjoining viscera, and upon the state of the air-passages, is sufficiently obvious. The difficulty of breathing proceeding from these sources may be either continued or remittent; but it never is, whilst the causes on which it depends are in existence, characterized by intervals of perfect ease. True asthma, however, presents intervals of healthy respiration; and, although repeated returns of the attack will generally induce some change in the organization of either the lungs or the principal organs of circulation, yet this is not uniformly the case; and moreover, an attentive examination of the thoracic viscera, in recent attacks, fails of detecting in them any appreciable change, particularly during the intervals between the paroxysms. The disease has even proved rapidly fatal during the attack, and yet no alteration adequate to account for the symptoms could be detected on dissection. Instances of this description have been adduced by Wichmann, (*Hufeland's Journ.* b. i. p. 18,) Parry, Georget, Andral, Laennec, and Guersent, and justify the opinions of those who have referred the disease chiefly to the nervous system. In some cases, after repeated returns of the attack, and when they have induced organic change, the intervals are less distinctly marked, consist of remissions merely, and the disease may at last pass into confirmed dyspnœa.

" *The structure of the air-passages and bronchi* evidently shews that these parts are susceptible of preternatural or spasmodic constriction. During 1821 and 1822, when engaged in some researches into the pathology of diseases affecting the trachea and bronchi, I was enabled distinctly to trace muscular fibres throughout those parts, both in man and in the lower animals. The disposition of those fibres in many of the lower animals, and the mode of their connexion with the cartilaginous rings, are peculiar, and beautifully adapted to guard against the contingencies to which they are liable from varying positions and habits of life. Upon those, however, I cannot here enter. About the same time that my attention was directed to this subject, (*London Med. Repository*, vol. xxii. p. 418,) the researches of Reisseissen, of Berlin, and of Laennec and Cruveilhier, of Paris, appeared; and the results, in respect of the structure of the bronchi and larger ramifications of the trachea, upon the whole, agree with what I have observed. It had been denied that the membraneous or any other part of the air-passages contain muscular fibres. But this was asserted chiefly by those who cannot believe that a part is muscular, unless the fibres are the same in appearance as those which enter into the composition of the muscles of voluntary motion. Other anatomists, who take a more comprehensive view of the conformation and functions of the muscular system, consider, with greater justice, that the muscles which are acted upon by the will form an order by themselves; and that there is another, and a very important order of muscular parts, which are not directly influenced by volition, but which contract, from stimuli acting on them, either immediately or mediately, and which present certain peculiarities in respect of the appearances of their fibres, of the mode of their distribution, and of the manner of their connexion with internal tissues and organs. Now the fibres which are discovered in the trachea, and traced to the smaller ramifications of the bronchi, are in every respect similar to other involuntary muscular fibres in their organization; in their connexion with a mucous surface, forming, in many respects, a tunic concentrically with the mucous coat; in being disposed in circular fibres, surrounding hollow tubes; and in being supplied entirely by ganglial or involuntary nerves. The disposition of the fibres therefore which are detected in the air-passages being altogether similar to that which obtains in other canals, the muscular structure of which is not disputed, as in the alimentary tube and urinary bladder; the organization of the fibres being also similar: their connexion to a mucous surface, and the circumstance of their being supplied with the same order of nerves, being at the same time considered: are we therefore to be surprised that agents affecting either the mucous surfaces thus related to them, or the nerves supplying them, should be followed with analogous effects to those which we observe after the action of agents directed to the mucous surface or nerves of the alimentary canal?

"*The lungs possess a vital power of expansion.* The structure of the air-passages, then, would lead us, independently of the results of observation, to infer that the circular fibres are liable to experience, with all other involuntary muscular fibres, a spasmodic constriction; and it evinces, particularly in the conformation of the cartilaginous rings with which the trachea and larger ramifications of the bronchi are provided, a marked provision against an inordinate continuance or degree of this constriction; the rings, by their permanent elasticity, acting as antagonists to the circular fibres, preventing extreme constriction, and at last overcoming long-continued spasm, particularly in those larger branches, the inordinate constriction of which might have the effect of excluding the air from a very large portion of the lungs. In the larger ramifications of the bronchi, the muscular fibres connecting the extremities of the cartilaginous rings are thus antagonised by these rings; but, in the smaller ramifications, where the rings cease to be detected even in the imperfect forms in which they there exist, and where the fibres are perfectly circular, the only provision which can prevent an inordinate constriction of those fibres is in the structure of the lungs themselves, which must necessarily undergo a change in bulk, and become more condensed by this constriction, in those parts, at least, to which the spasm extends; unless we believe that the lungs, like various other organs, are endowed with an expansive power, a power which physiologists and pathologists have too much overlooked in their exposition of the healthy and morbid actions of the animal economy.*

"The mechanism of the expansive power is so little understood, and generally so insufficient for the explanation of this phenomenon, that we must refer chiefly to the vital actions of the part, which must necessarily depend on the energies of the body generally. The expansile action of the penis, nipple, heart, uterus, &c. cannot be explained by their organization only: it is manifested to us only during life, and the perfection as well as imperfection of this action are always accordant with the degree of vital energy with which these organs are endowed.

* "That the lungs, however, really possess this property, may be inferred from the permanent elasticity of their structure, which continues for some time after death; and which is still more marked during life, as shewn by exposing the lungs of a living animal. This state may be with propriety called the vital expansibility of the lungs, inasmuch as the degree of this state is chiefly dependent upon the vital energy of the system, and partly on the peculiar organization of the lungs themselves."

"I have long since had occasion to remark that the motions and functions of the lungs (*Physiological Notes, &c. to* M. Richerand's *Physiology*, 2d edit. p. 628,) have been too generally and exclusively referred to the mechanism of the respiratory organs, and to chemical changes produced in the lungs, to the neglect of a much higher influence, always controlling, modifying, or altogether changing, the subordinate powers to which their functions have been thus referred. That the vital energies of the frame are most powerfully exerted in the lungs, through the medium especially of the organic nerves with which they are provided, must be evident to all who will contemplate the nature and extent of the changes constantly taking place in these organs upon the blood circulating through them; and the relation which subsists between their functions and the vital energies of the system generally. Now it does appear to me that there exists a vital expansion of the lungs, independent of that which they experience from atmospheric pressure, and from following the dilated parietes of the thorax during inspiration. In experiments upon living animals, where the walls of the chest have been opened, the lungs are observed to swell and contract alternately. This fact, which was first insisted upon by M. Roux, (*Mélanges de Chirurg.* p. 87,) has since been duly appreciated by Prus, Laennec, and a few others. Even in cases where the portion of lung has protruded itself after a wound of the chest, (a circumstance which could only occur from active expansion of the lung itself,) this portion has been, although thus unnaturally placed and subjected to the pressure of the atmosphere, observed to dilate during inspiration. The not infrequent occurrence of ossification of the cartilages of the ribs in old persons, and consequent perfect immobility of the ribs, even without any evident dyspnœa, furnishes another proof of the inherent expansibility of the lungs; for, without having recourse to this vital property, we cannot explain the performance of the actions of inspiration and expiration by the diaphragm alone.

"This vital property therefore, with which the lungs, in common with some other organs, seem to be endowed, together with the disposition and elasticity of the cartilaginous rings of the bronchi, furnishes an antagonising force to the unnatural constriction of the tubes, from spasm of their circular fibres; and, while it serves to explain the natural functions of the organ, with their modifications from the various influences to which this property is subjected, is one of the sources to which we are to impute some of the diseases, and more especially the one under consideration, to which the lungs are liable." (P. 135.)

Having thus shewn, from the structure of the air-passages, that they, in common with all other hollow tubes of the body, admit of spasmodic constriction, and that they also present a provision against the undue extent or continuance of this state, Dr. C. further remarks, that a close observation of the manner of performing respiration is sufficient to convince us that they frequently experience this state, owing to the operation of certain causes acting either directly on the mucous surface of the tubes, and impressing the nerves terminating in it, or originating in, and irritating the nerves themselves, either at their origins, or in their ramifications and connexions.

*Symptoms and history of Asthma.* The premonitory symptoms of this disease are langour, sickness, flatulency, and other dyspeptic disorders; heaviness over the eyes, and headach; uneasiness and anxiety about the præcordia, with a sense of fulness and straitness in this region and in the epigastrium. In some cases pain is complained of in the neck, with uncommon drowsiness and stupor. Costiveness is often a precursory symptom.

"The *invasion* of the attack of *spasmodic asthma* is generally soon after midnight, or about one or two in the morning, and during the first sleep. The patient wakes suddenly from a sense of suffocation. He feels a most distressing tightness at his chest, with great anxiety, difficulty of breathing, and impediment to the free admission of air into the lungs. He assumes with eagerness the erect posture, and cannot bear the least incumbrance about the chest. The breathing is wheezing, interrupted, and laborious. The shoulders are raised, the elbows directed backwards, and every effort made to enlarge the thorax. Owing to the interrupted circulation through the lungs and heart, the countenance, which was at first pale and anxious, becomes, particularly in plethoric habits, red or bloated, and covered with perspiration. The eyes are prominent, and the conjunctiva injected. A considerable quantity of pale urine is usually passed at the commencement, or previous to the accession, of the paroxysm; and the lower extremities are usually cold. The pulse is generally accelerated, weak, irregular, and often intermittent. During the fit, the patient has commonly an instinctive desire for cool fresh air, which always revives him. A small or close room is offensive, and all warm substances given internally increase the flatulency of the stomach and bowels, and aggravate the symptoms. When the fit has continued from half an hour to one, two, three, or even four hours, it leaves the patient; and his respiration, pulse, and feelings assume their natural state.

"This is the common course of a first and moderate attack of the disorder. Sometimes the patient has but one such fit; but more generally a slight constriction of the chest is felt through all the succeeding day, and the paroxysm returns at the usual period of the night; and this continues for three, four, or even seven days; when the patient is at last altogether relieved from the attack. The disease may be suspended for a month, or several months; but it is liable to recur from changes of air, errors of diet, and from the operation of the other causes productive of it.

"In some cases the attack is more severe from the commencement, and continues, with slight remissions, for several days, accompanied by a harsh suffocative cough, great distention of the abdomen from flatus, and more or less of the symptoms which characterise the complaint in the severer states resulting from repeated attacks.

"When asthma once seizes on the system, it seldom fails of recurring, though the intervals between the paroxysms are of very uncertain duration. In many cases it recurs periodically every ten days or a fortnight. Sometimes the attack returns at the full and change of the moon, or at one of those periods only. It has been observed to recur in females just after the menstrual discharge, or to precede this evacuation. Persons who have become subject to the disease seldom escape an attack in the spring and autumn.

"After repeated seizures, the disease often assumes the most violent and distressing features; the difficulty of breathing in the fit amounts to the utmost degree, and is attended with the greatest tightness over the whole chest, the patient feeling as if he were bound with cords. His anxiety at this period is inexpressible, and he labours in respiration as if every moment would be his last. Severe vomiting also frequently occurs. The matter discharged is slimy and frothy, or of a greenish yellow colour. He is subject to palpitations and faintness; and cool fresh air becomes absolutely necessary. About this period a loose stool sometimes takes place. The eyes are prominent, the face sometimes pale, sometimes high-coloured, bloated, or livid: the nose and ears are cold; the face, neck, and chest, covered with perspiration. The pulse is generally extremely weak, irregular, and even intermitting; there is often much difficulty of swallowing. The patient can scarcely speak, cough, or expectorate, and the stomach and bowels are much distended with flatus. As the paroxysm abates, the cough becomes freer, and is attended with the expectoration of a little viscid mucus; and, in proportion as the cough and expectoration increase, the distressing symptoms abate; this evacuation, which had been retained by the spasm of the air-vessels, indicating a solution of the spasm and a freer access of air to the cells of the lungs. An easy and free expectoration, particularly if it be accompanied with softness and moisture of the skin, and a sediment in the urine, is a certain indication of the subsidence of the attack. Sometimes, when the paroxysm is unusually long, the patient experiences only a single occurrence of it during the attack." (P. 137.)

The *humoral* form of asthma is generally gradual in its accession, and attended by extreme oppression, or suffocative cough, and a copious secretion and expectoration of mucus from the commencement of the seizure. It is sometimes the consequence of repeated attacks of the preceding variety; and is generally more severe and of longer duration than it, owing to the accumulation of viscid mucus in the air-vessels conspiring with the spasm it occasions to aggravate the symptoms. There are also less perfect intervals of ease in this form of the malady than in the spasmodic. After the subsidence of the patient's sufferings during the first night of the attack, and while the expectoration is easy and copious, the lungs still continue irritable throughout the day, and the respiratory function embarrassed from the slightest causes. At the approach of night, the fit re-commences with the usual symptoms, and the night is passed nearly as the former. On the third day the remission is more complete; there is some additional expectoration, and bodily motion is performed with less distress, but still with great inconvenience. After the paroxysm has been renewed in this manner for three or four nights, or for a longer period, sometimes for several days, or even weeks, the expectoration and cough are more easy and free, the daily remissions become more perfect, and the strength of pulse and vigour of action increase.

"When the chest is examined by the ear or stethoscope, the sound of respiration is weaker during the fits than in the intervals, but it is seldom altogether suspended in certain points of the chest; it is attended by a sonorous rattle, flat or sibilous, imitating the chirping of birds, the note of a violoncello, or the cooing of the wood-pigeon. With this there is frequently intermixed a mucous rattle; but this conveys the impression of being produced by a thinner fluid than the mucus of common catarrh. In the intervals of the attacks, these various species of rattle exist, but in a much less degree. The respiratory sound is louder than during the paroxysms: sometimes it is almost puerile. If the complaint have occasioned dilatation of the bronchi, the respiration assumes more or less the character of the variety called bronchial; in all cases it varies in intensity at different points of the chest, and these points change their situations from day to day (Laennec.) The chest generally sounds well, throughout the attack, upon percussion." (P. 137.)

The humoral form of asthma is often consequent upon repeated attacks of the spasmodic, but the latter may also occur after the former; or the attacks in some persons present an evident complication of both forms of the disease. Disorders of the stomach and bowels are common in asthmatic patients. In females, menstruation is generally irregular, and an attack often precedes the period of the menstrual discharge. An attack of asthma terminates either by a return to the healthy functions, or by inducing further lesion, in which it either disappears or becomes complicated; and in death. On each of these terminations Dr. Copland offers some excellent practical comments.

"Although the paroxysms of asthma frequently terminate in a return to the healthy functions, a perfect immunity from future attacks can rarely be procured. Yet these attacks may be frequent, severe, and of long duration, recurring for a long series of years; the patient, notwithstanding, arriving at a very advanced age, before a fatal issue takes place. But they often produce the following organic lesions.

"The most common consequences of the disease to which I may now advert, are, chronic inflammation and dilatation of the bronchi, the different forms of emphysema and œdema; of the lungs; hæmoptysis; tubercular formations, with which asthma may also be associated from its commencement; enlargement, and dilatation, &c. of the cavities of the heart; effusions of fluid in the pleura or pericardium; and wasting of the heart, or polypous concretions, within its cavities. As the reader will find all those lesions treated of under their distinctive heads, I shall here only remark respecting them, that, when they supervene to asthma, many of the distinctive characters of this disorder entirely disappear in those of the superinduced disease, and the lesions of the respiratory functions assume the distinctive features of chronic, continued, or remittent dyspnœa. Severe attacks of asthma may also terminate in congestions or effusions within the head, giving rise either to epilepsy, coma, or apoplexy.

"It was already remarked that auscultation and percussion furnished merely negative information in the different forms of asthma. But this information is still important, inasmuch as it intimates the non-existence of any of the foregoing organic changes; and, when they do exist, those means of diagnosis enable us not only to recognize them, but also to ascertain with precision their nature, progress, and extent, and thus to form an accurate diagnosis and prognosis in respect both of the primary disease and of the consecutive organic changes.

"When the disease ends in death, this event is brought about generally by superinducing some one of those changes already referred to as terminations of the disease, or of those lesions with which it is frequently associated. Death may however occur, but much more rarely, from the severity of the attack; the requisite changes not being effected on the blood by respiration, owing to the obstructed state of the air-vessels, either from spasm or the accumulation of viscid mucus, or from both, whereby the nervous centres are supplied with blood unsuitable to their functions, and the heart ceases to contract with sufficient energy to preserve the circulation in a requisite state of activity through the lungs and brain.

"*The appearances after death* may be inferred from what has already been stated. These appearances are rather the consequences of the disease, than the disease itself; for it is seldom that we have an opportunity of examining the body in recent and uncomplicated cases of asthma. Where however this has been done, the lesions, even when they have been detected, have been insufficient to account for the disease. Willis records a case of protracted asthma, in which no morbid appearances could be detected; and similar cases have occurred to Laennec, Andral, Cruveilhier, Bouillaud, Joly, and others. Ferrus, after extensive experience, states that he has been unable to detect any lesions which can be attributed to uncomplicated asthma. The changes which have been noticed therefore by authors are to be viewed chiefly as accidental occurrences, or associated maladies, and perhaps more frequently as the remote results of repeated or protracted attacks. The appearances usually observed in fatal cases are the same as have been described." (P. 138.)

*Varieties of Asthma, and of their Pathology.* By some pathologists this disease has been divided into many forms, several of which must be regarded as more fanciful than practically applicable. Dr. Copland admits only the idiopathic and symptomatic forms; and he divides the former into the nervous, the primarily spasmodic, and the pituitous or humid asthma.

Nervous asthma was first accurately described by Laennec, who pointed out the difference between it and the forms depending on spasm of the air-tubes. It is characterized by anhelation, from a feeling of want of a more complete respiration than the patient enjoys, the pulmonary expansion distinctly taking place with promptitude, completeness, and uniformity, so as to furnish a general puerile sound on auscultation, usually accompanied with a slight cough, and with a free mucous expectoration. In this variety no spasm seems to exist in the smaller air-vessels; for the whole tissue of the lungs is dilated to its full capacity, and with unusual promptitude and completeness, so that the puerile respiration is heard in every part of the chest; whereas, in the other varieties, the respiration is generally somewhat more indistinct than in health. The pulmonary expansion evidently takes place completely and rapidly in all the air-cells, and yet the patient feels the want of a more extensive respiration than he enjoys; and the lungs, though dilated to their utmost, have not capacity enough to satisfy the wants of the system. This affection is common in persons affected with chronic mucous catarrhs, attended by a copious and easy expectoration; but even in them, during the severest attacks, the completeness with which respiration is performed is quite astonishing.

"In this form of the disease it is not in the small air-tubes that we are to look for its proximate cause, but in the trachea and large bronchial trunks, and particularly in the nervous influence itself; and this will equally hold good even if we adopt the chemical theory of respiration, and refer the affection to an extraordinary want of oxygen in the blood, arising from impeded function of the respiratory mucous surface, owing to the mucous secretion covering it. M. Laennec believes, as this species occurs only in persons affected with chronic mucous catarrh, that it can never amount to asthma without the catarrhal complication. Adults and old persons, he remarks, who have puerile respiration without catarrh, are not, properly speaking, asthmatic, but they are short-breathed; and dyspnœa is induced by the slightest exertion, though, when sitting still, they frequently experience no oppression whatever.

"This variety may be considered as depending upon a temporary augmentation of the want of the system for respiration, occasioned most probably by some unknown modification of the nervous influence; and apparently consisting in an expansile action of the lungs increased much beyond the healthy standard. But here a question suggests itself, viz. can this augmented action of the lungs be owing solely to the state of this organ, or is it associated with, or partly depending upon, increased activity of the respiratory muscles, particularly the diaphragm? M. Laennec states that it cannot be produced at will by a full inspiration; and therefore infers that this state of the lungs is a primary condition of them, and not depending on increased respiratory efforts.

"From this consideration I am led to infer that, although the vital expansile action of the lungs may be increased in this variety of asthma, it is accompanied with, and much assisted by, augmented activity of the diaphragm, which performs its office more promptly and completely in this variety of asthma than in any other; that, instead of the disease being characterised by spasm of the smaller ramifications of the bronchi and air-cells, as in the second variety of asthma, the air penetrates more fully into them than usual; and that, if any spasm exists, it is limited to the trachea and large bronchial tubes; the exalted state of expansion of the lungs, and of function of the diaphragm, being an effort to counteract this morbid condition of the large tubes, and to supply the wants of the system by a more forcible inspiration, the increased rapidity with which the air is thereby made to pass through the strictured canal making more than amends for the diminished caliber of the passage. This form of the disease is frequently *symptomatic* of nervous affections, particularly of hysteria, when the globus hystericus affects the state of the trachea, and of various diseases, in which the blood is imperfectly changed in its circulation through the lungs. But when thus symptomatic, it is often slight and evanescent." (P. 139.)

*Spasmodic Asthma.* In this variety the paroxysms are sudden, violent, and of short duration, and attended with hard spasmodic constriction in the chest; slight, dry, and difficult cough, and with a scanty expectoration, occurring only towards their close. Here the ramifications of the air-tubes, and perhaps the air-cells themselves, seem to be unnaturally constricted. By auscultation it may be ascertained that there is an imperfect entrance of air into the air-cells.

The paroxysms of this form may be short; but the disease may continue for a long time, and to a certain extent become habitual. Sometimes, in consequence of the violence of the spasmodic struggle, rupture of one or more of the air-vessels or cells takes place, from the violent action of the inspiratory muscles on the one hand, and the unyielding state of constriction of the air-vessels on the other; and emphysema of the lungs is superinduced, forming one of the most common lesions found upon dissection of fatal cases, and, in the opinion of some pathologists, the proximate cause of the disease.

The *common*, or *humid asthma*, is marked by a gradual accession of the paroxysms, which increase in severity, are protracted, and attended with heavy and laborious constriction of the thorax, severe suffocative cough, and with expectoration, often commencing early, at first viscid and scanty, but becoming copious, and affording relief.

"The chief characteristic of this variety of asthma is the copious discharge of viscid mucus accompanying it. But the questions with several modern pathologists have been, whether the phenomena of the disease are to be imputed solely to the accumulation of this fluid in the air-passages, or in part only; and whether spasm of those passages also exist in conjunction with an increased secretion of mucus, or not. I believe that an attentive observation of the phenomena of the disease, with the assistance of auscultation and percussion, (which, however, occasionally furnish but little information, and that of a negative description, in this disease,) will lead to the inference that it depends upon both those morbid states. The limits of our inquiry are too narrowed to the question of the priority of their existence, and the relation which the one holds to the other. As to these points, it may be remarked, that the early occurrence of expectoration, as well as its abundance, forbid the inference that the production of viscid mucus is the consequence of relaxation of the spasm; whilst they favour the idea that the spasm is occasioned by this secretion in the irritable and morbid air-tubes; the severity and duration of the paroxysms being occasioned by these double states of disease,—an abundant secretion of viscid mucus in, and a spastic constriction of, the air-passages.

"But it may be further inquired, are not those morbid changes the effect merely of a certain condition of the air-passages, still more intimately connected with the disease than they are? I do not deny the possibility of lesions antecedent to those now specified; but the difficulty of ascertaining their exact nature must be conceded. It would certainly be advantageous to obtain this information, inasmuch as on it would be based the means of cure which might be employed early in the disease. That it is not inflammation is proved by concomitant and symptomatic phenomena, by the course of the paroxysms and of the disease, by the terminations usually characterizing it, and by observation of the juvantia and lædantia during its progress. It seems, however, extremely probable that the morbidly increased secretion and spasm are preceded by a congestive state of the mucous respiratory surface; this state disposing to the spasm, and being, as well as the spasm itself, at last relieved by the copious effusion of mucus; the mucus first effused tending however for a time to increase the spastic constriction of the air-passages, and the consequent struggle of the respiratory muscles to overcome it, and to procure a fresh supply of air in the lungs. This antecedent state of vascular turgescence of the mucous surface of the bronchi in asthma, is perhaps most marked in that form of this variety in which little or no expectoration accompanies the cough, at least, early in the attack; and which, from this circumstance, and the causes which induce it, has been called the *dry* catarrhal asthma.

"If it be still further asked, to what cause are we to impute this congestive state of the respiratory surfaces? I can only answer, to a certain primary change of the vital energy of the organic nerves supplying the blood-vessels, and actuating the muscular fibres of the bronchi; and hence, as the morbid changes of the circulation, secretion, and caliber of the air-passages, are merely effects of one cause —of a previous change of the vital manifestations of the nerves of the organ, it becomes of the utmost importance to ascertain the nature of this primary change with as much accuracy as possible, in order that remedial agents may be directed with precision to its removal; but the prosecution of this very interesting topic falls under another division of my subject. In estimating, however, the nature of this, as well as the other varieties of asthma, the difficulties opposed to expiration by the spasm of the air-tubes, and the accumulation of viscid mucus in them, have been too generally overlooked, in our eagerness to ascribe all the morbid phenomena to impeded inspiration. But I believe that the disease, particularly this variety of it, is as much occasioned by the obstacle these states of the air-passages present to free expiration; the air, by the greater power of the inspiratory over the expiratory muscles, being drawn in sufficient abundance into the lungs, from which it is imperfectly expelled. From this circumstance the lungs are often kept in a state of inordinate dilatation, and the respiratory muscles excited to convulsive actions, occasioning dilatation or rupture of the air-cells, and consequent emphysema of the lungs. In the more advanced stages of the disease, in old and debilitated subjects, this struggle to dilate the thorax still further, proceeding from the wants of the system for respiration, and to expel the air from the lungs through the obstacles placed in its way, generally terminates unfavorably to the latter part of the respiratory actions; consequently, expectoration is impeded or suppressed, and life is terminated, with the air tubes and cells, and even the substance of the lungs loaded and infiltrated with mucus, air, and serum. It is in this state that active stimulants and emetics, by rousing the energies of the frame, and by exciting the expiratory efforts during the process of vomiting, prove so frequently beneficial.

"This form of asthma may be partial, affecting one lung only, or one more than another; but it is more commonly general; and in some constitutions, particularly in aged persons, and when it has supervened to repeated attacks of catarrh, the quantity of viscid mucus expectorated is very great.

"Its *anatomical characters* are, slight swelling, or thickening, and softening of the mucous membrane, with a slight appearance of redness in parts, and with marked congestion, and purplish tint of portions of this surface, in the more severe or protracted cases. Sometimes these lesions are accompanied with slight œdema of the membrane, and the development of miliary tubercles in the lungs.

"As the majority of cases of this disease is characterized from the commencement by copious expectoration, it becomes a question how far it deserves to be considered as a variety of asthma; but, taking all its phenomena into consideration, particularly the spasm of the air-passages, and convulsive action of the respiratory muscles, as well as the circumstance of it having been usually considered as a species of asthma, and the difficulty of arranging it otherwise, I was unwilling either to assign it a different place, or to make it a distinct disease, to which it scarcely can lay claim. M. Laennec has placed it among catarrhal inflammatory affections of the bronchi: but I conceive that it is seldom inflammatory, either in its origin or progress; and that, although occasionally commencing in, and always aggravated by catarrh, it is not necessarily a catarrhal disease. Besides, inflammations of the bronchi and catarrhs are not identical affections, although the latter frequently pass into the former.

"But, besides these considerations, many of the phenomena essentially characteristic of asthma always attend it, to a greater or less extent. Upon an attentive examination, however, of the chest of a person afflicted with this affection, by auscultation and percussion, these phenomena are found to vary, in different cases, or even in the same case, at different periods of the attack; yet they are essentially the same as those which mark the preceding varieties, although not so evident to the senses as in them, inasmuch as they are obscured by a more prominent symptom,—the copious mucous secretion and expectoration. Sometimes it is manifest that certain parts of the air-tubes are differently, or even oppositely, affected at different periods of the attack. When the viscid mucous secretion proceeds from, and is still present in, the smaller ramifications of the air-vessels, this condition, together with some degree of spastic constriction of their circular fibres, either in a part only, or more or less throughout the organ, occasion many of the symptoms which characterize the second, or spasmodic variety of the disease. But, in proportion as the secretion rises to the larger air-tubes, and leaves the smaller ramifications clear; or when the mucous secretion proceeds chiefly from the former parts, and excites, or is accompanied with, spasms of these canals, but not to the extent of preventing the passage of air into the parts of the lungs which they supply; these parts generally expand freely, owing to the vital activity of the organ, the wants of the system for the changes effected on the blood by respiration, and the active contraction of the inspiratory muscles during the convulsive efforts of the paroxysm. Hence the part of the lungs thus affected generally furnish the puerile respiration, and a clear sound on percussion, with a full and prompt performance of the inspiratory actions; phenomena characteristic of the first, or nervous form of asthma." (P. 141.)

*Symptomatic asthma* may arise from various organic lesions of the thoracic and abdominal viscera; and sometimes it is the consequence of lesions affecting the medulla oblongata and spinal cord, of hypochondriasis, and diseases of the colon and rectum.

"Upon taking a review of the causes of this malady, we shall perceive that it may be occasioned, like several other chronic diseases of the respiratory organs, 1st, by whatever lowers the vital energies of the frame, particularly as they are manifested in the lungs, and increases the susceptibility of the organ to the impression of external agents, or to internal morbid associations; 2d, by mental or moral states deranging the nervous influence, actuating the respiratory and circulating organs; 3d, by agents which disturb the equilibrium existing between the cutaneous and respiratory functions; 4th, by causes acting, during respiration, directly on the seat of disease, either by depressing the vital and nervous influence of the organ, or by irritating its mucous surface, and thereby exciting its fibrous structure to undue contraction; 5th, by causes acting during respiration, especially aërial vicissitudes and states which modify or impede the respiratory functions, and favour congestion of the pulmonary mucous surface, or of the substance of the lungs; 6th, by whatever impedes the action of the respiratory muscles, or embarrasses the motions of the parietes of the chest; 7th, by lesions of the circulating organs deranging the circulatory function of the lungs or heart; 8th, by the extension of irritation from adjoining viscera or parts; 9th, by the destruction of the equilibrium between absorption and excretion; 10th, by the transference of morbid action from other parts of the frame; 11th, by affections of the respiratory nerves and plexuses, either at their origins or in any part of their distributions. Hence the propriety of dividing asthma not only into the *nervous*, *spasmodic*, and *humid* varieties, but also into two divisions, as respects its relations to its causes, and to other diseases, viz. into *idiopathic* and *symptomatic*." (P. 146.)

*Proximate cause.* Most writers have referred it to spasm of the bronchial tubes. By some pathologists the disease is regarded as altogether symptomatic of organic changes seated chiefly in the heart and large vessels; but, admitting that this is occasionally the case, Dr. Copland conceives that they substitute the effect for the cause; and his opinion is not only corroborated, but confirmed, by many post-mortem examinations, in which no such changes were found. He concludes that asthma depends on a preternatural or spasmodic constriction of the air-passages, accompanied, in many cases, especially the humoral or catarrhal variety, with turgescence of the vessels of the lungs, particularly those supplying their mucous surface, and an increased secretion of mucus.

*Treatment.* The physician has two objects to fulfil: to shorten or alleviate the fit, and to prevent its return. In treating the fit of asthma, the practitioner must bear in mind certain circumstances which materially influence the choice, combination, and extent of the means to be employed. The duration of the paroxysm; the age, temperament, and habit of body of the patient; the period he has been subject to the disease; the frequency of the attacks, and the particular form they assume; the state of health in the interval; and the presence or absence of concomitant, functional, or organic lesions of the lungs, heart, and digestive organs, are altogether of the utmost importance to be known. Bleeding in young and plethoric subjects will be required; but it will seldom do more than relieve urgent symptoms. It will rarely, if ever, put a stop to the paroxysm, and should always be practised with much caution. Antispasmodics, anodynes, and narcotics, are chiefly beneficial in the first and second forms of the disease; and in the third, when attended with severe convulsive cough. Camphor is highly spoken of by Dr. C.: it is, he says, when judiciously exhibited, applicable to nearly all the forms and complications of the disease. It should be given in doses of from three to ten grains. If the symptoms of the paroxysm were very severe and urgent, we should prefer æther, as more prompt in its effect. Ipecacuanha, besides its good effects as an emetic, is one of the best medicines that can be resorted to in asthma, and is suited to all the states of the disease, particularly when judiciously combined with other remedies. Dr. Copland has found much benefit from the distilled laurel-water, or prussic acid, during the paroxysm. He prefers the compound tincture of opium to morphia. Loebel praises very highly a combination of hyoscyamus with infusion of valerian. Belladonna has been found serviceable in combination with camphor, valerian, or assafœtida.

"Narcotics are most quick in their operation when their vapour or smoke is inhaled into the lungs. Their effects are longest delayed when they are applied to the external surface; unless the cuticle has been previously removed, as in the 'endermic' method of medication. The inhalation of the vapour of certain of this class of remedies, either alone or in conjunction with some volatile vapours, is one of the most certain and quick modes of obtaining relief in the asthmatic paroxysm.

"Stramonium is one of the best remedies that can be prescribed in the spasmodic form of asthma. It is principally used by smoking it as tobacco. During this process, the patient may either draw a portion of the smoke into the lungs, or swallow some of it, or the saliva which has become impregnated with it. Stramonium is very advantageously smoked along with aniseed, or with a small portion of tobacco." (P. 148.)

Lobelia inflata has been much employed in America, and several English practitioners of celebrity think well of its powers in asthmatic cases. In several cases in which we have ourselves given this remedy, we have been well satisfied with its good effects. Tobacco smoking is only productive of marked benefit, Dr. C. remarks, when it excites a free expectoration; and next, and perhaps equal to smoking, is the inhalation of simply emollient, or of medicated vapours, into the lungs.

*To remove viscid phlegm, and to prevent its formation,* expectorants, as squills, ammoniacum, and senega, are generally, and not always with proper caution, prescribed.

"The good effects of these medicines in certain manifestations of asthma cannot be doubted; but I have seen them productive of much mischief in several cases in which they had been employed. It should be kept in recollection that they are amongst the most active excitants of the respiratory mucous surfaces we possess, and extremely apt to change active congestion of the bronchial lining into inflammatory action, especially in young, plethoric, or robust subjects; and, by their effect upon the expectoration—particularly by increasing it, rendering it thinner, less viscid, and more readily expectorated, to occasion a deceptive appearance of benefit, even when they are increasing morbid action, with all its ill effects. In relaxed and leucophlegmatic habits, however, or when the expectoration is viscid, and excreted with difficulty, the skin cool, soft, and moist; the pulse soft, slow, or weak, and the urine scanty; these medicines may be given with great benefit (see F. 66, 67, 74, 350): but when the pulse is either hard, quick, or full; or the expectoration at all puriform; they cannot be exhibited without risk. They will often, doubtless, even in cases of active congestion of the respiratory mucous surfaces, afford real benefit, by exciting the capillaries to secretion, and thereby unloading them; but they may as readily kindle up inflammatory action. When combined, however, with antimonials, refrigerants, diuretics, or anodynes, the risk of mischief from them in doubtful cases is much reduced." (P. 149.)

Emetics are amongst the most promptly beneficial remedies that can be resorted to during the paroxysm, to remove both phlegm and spasm. Nauseants and diaphoretics are allied to emetics, and are often of service at the commencement, or shortly before the fit.

*Refrigerants.* Nitrate of potash is the most useful in conjunction with camphor, ipecacuanha, and hyoscyamus, particularly in the humoral variety of the disease. Loeffler recommends cold epithems to be placed on the chest in the spasmodic form of the disease; and several continental writers advise clysters of cold water to be administered, when asthma seems to be connected with hysteria. Revulsion and derivation, by the ordinary and well-known means, must be had recourse to when asthmatic attacks are dependent on metastasis of gout, rheumatism, &c. During the paroxysms, flatulence is often a troublesome symptom, and must be removed by carminatives. Warm coffee frequently relieves the fit of asthma; and Dr. Copland tells us that he has found green tea answer the same purpose.

For the treatment required *during the interval of the paroxysms,* we must refer to the article itself.

The more frequently we refer to Dr. Copland's Dictionary, the more reason do we find to approve of the manner in which it is executed, and to admire the close yet clear style in which, in a brief space, he has contrived to give the principal facts upon all subjects that can interest the practical reader.

---

*Clinical Lectures on the Contagious Typhus, epidemic in Glasgow and the Vicinity, during the Years* 1831 *and* 1832. By RICHARD MILLAR, M.D., Senior Physician to the Royal Infirmary, and Regius Professor of Materia Medica in the University of Glasgow.—8vo. pp. 184. Brash and Co., Glasgow; Black, Edinburgh; Longman and Co., London.

TYPHUS is a subject of commanding importance, and, notwithstanding our attention has been so frequently directed to this disease by the numerous practical and controversial essays on its origin, treatment, and prevention, which almost daily issue from the press, it is one to which we always turn with a fearful interest, as to the records of the deaths of thousands. In this metropolis, from which typhus is never absent, its ravages are in some measure checked by the prompt relief afforded, and the extensive liberality of the opulent; yet even amongst us its fatality is occasionally great. But, in other towns, and in many places, where such ample means are not always immediately at command, its ravages are dreadful. In Ireland typhus is unhappily most prevalent. Dr. O'Brien states, that in one year there were received into the different hospitals of the city of Dublin alone no less than 41,775 patients with fever; and, adding to these the extra hospital cases, the whole number in the metropolis did not, within the time specified, fall short of 50,000.

"In Ireland alone," says our author, "it is computed that at least a million of the inhabitants, within the last twenty years, have, from this source, suffered the infection of typhus. Even bodily fatigue and anxiety of mind are found sufficient of themselves to excite the distemper, as we see in private families, where individuals, from duty or affection, have been induced to watch over, daily and nightly, the course of the disease in some near or beloved relative. Such persons, from mere exhaustion, very generally catch the fever; and I think I have observed that in these cases it for the most part terminates fatally." (P. 6.)

From time to time this disease becomes epidemic, raging more especially among the poorer inhabitants of large towns, few of whom remain many years together without a visitation. In the years 1831 and 1832, Glasgow and its vicinity became subject to the scourge; and an account of the disease as it manifested itself in that place, with some general remarks on the history, symptoms, and treatment of typhus, form the subject of Dr. MILLAR's clinical lectures.

The volume is divided into two sections, the one *practical*, the other *controversial*. In the first, the diagnosis, causes, prognosis, and treatment of typhus fever, are described; in the second, many of the theories and speculations of the past and present time are discussed; and the work concludes with some general results drawn from the reports from various hospitals, and a few remarks on Prophylaxis.

As the controversial section, although it occupies nearly half the volume, leaves the disputed points in the same undetermined state in which it found them, we do not propose to analyse its contents. The practical section is much more to our taste; for it contains a clear and sensible account of what is seen in the disease called *typhus*, originate how it may, and of what is to be done for its removal, whatever the mode in which the means may act. Let it not, however, for a moment be supposed that we undervalue theory, properly so called, or even speculation; when the one is a contemplation or enlarged and liberal view of truth, and the other springs from facts already well ascertained, being as it were the harbinger of future discoveries; but we do utterly abhor those speculations which reflect the vagaries only of an author's wayward imagination, and those theories (falsely so called) in which *facts* are made to suit *opinions,* as travellers were stretched, or their limbs curtailed, to make them fit for Procrustes' iron bed.

The anti-contagionists will object to the title Dr. Millar has chosen, as being a prejudgment of one of the most controverted points. Our opinion upon this subject is already on record; and, as we do not find anything new in our author's arguments, we shall not deviate from our resolution of confining our remarks to his practical section.

The conclusions which have been arrived at by a physician or surgeon who has had extensive opportunities of studying disease, form some of the most valuable parts of medical science: for, although the records of facts upon which opinions are founded are often considered, and with propriety, still more valuable, yet they of necessity are so numerous, and, even when the most complete, are so comparatively imperfect, that few persons are able thoroughly to understand, and fairly to estimate the value of written evidence; and none so capable of attaching their due importance to symptoms as they arise as an intelligent and unprejudiced observer.

Cullen's definition of typhus, and the refinements of the French nosologists, we need not dwell on here; they are too well known to all our readers. But, in considering the prognosis which should be given, our author places some of the symptoms in a new light, and puts others in very striking points of view. Speaking of the pulse, he records a case which should be another warning never to relax exertions, even in the most desperate and apparently hopeless cases. "While there is life there is hope."

"A very feeble pulse, though dangerous, is not necessarily fatal, provided the accompanying symptoms are not particularly urgent. I have repeatedly restored patients, where the circulation in the arm was a mere thread, or flutter: in the following remarkable instance it was extinguished altogether. Going my rounds, in the hospital, one day, I came to a bed where the occupant seemed in the last extremity. Among other deadly symptoms, the pulse at the wrist was entirely gone. So hopeless indeed did I think him, that I passed on without any prescription. About an hour afterwards, however, some signs of vitality appearing, my clerk luckily conceived the idea that something might be done by powerful stimulants, and as deglutition remained, he plied him with large and repeated doses of wine, spirits, and sulphuric ether. The experiment succeeded. The pulse soon rallied, though feebly; the previously threatening symptoms declined by degrees; and at my visit to the hospital next day, instead of dead, I found the man lying with his powers of life revived, the pulse beating respectably firm, the other functions improved. Not the slightest relapse occurred. In two days the patient was out of danger; after the usual time he was dismissed cured. This is no doubt a solitary instance, but it shows that we should never despair." (P. 14.)

The following cautions with respect to the state of the tongue and the appetite are well worthy attention.

"A return of the tongue to its natural state is always reputed a favorable sign, and the amelioration generally takes place first at the sides; the mark, however, is not always infallible. Occasionally the tongue recovers itself, though the other symptoms do not yield, while, on the contrary, it sometimes continues foul or dry, after the other morbid appearances recede. In the first instance, we look for a return of appetite, but are disappointed; while in the second, some desire of food returns, though unexpected. When the disordered state of the tongue thus survives the fever that gave it birth, it can no longer be considered as an index, or guide, to the general condition of the system: it is a mere insulated sequela, in its nature purely topical, and to be treated accordingly."* (P. 15.)

"With respect to the revival of appetite, it is needless to say how gladly it is to be hailed, provided, at least, it be coincident with other favorable signs. A curious example to show the necessity of this prognostic limitation occurs in delirium. During this state, patients are not unfrequently observed, not only to receive food unconsciously, when presented to them, but to swallow it with all the apparent greediness of hunger. Hence nurses are often imposed upon. If you ask a nurse how the sick person takes his food, she will reply perfectly well: but if you find that at the same time the pulse, the tongue, and sensorial functions, have attained no improvement, you will then detect the fallacy, and discover that this reported restoration of appetite is merely illusory, and is neither more nor less than a symptom of delirium, or of the hallucination of mind that forms so prominent a symptom in our low contagious typhus." (P. 16.)

The symptoms of derangement in the sensorial functions, and the prognostic signs deducible therefrom, are very graphically drawn. They have evidently been often seen and considered before they were described.

* "A curious circumstance with respect to the tongue may be often noticed in patients labouring under delirium. If you desire them, in this state, to put it out, they readily obey; but, unless you urge them frequently, they often neglect to draw it back. The functions of memory, and of the association of ideas, are here impaired, along with other sensorial powers. The delirious person almost instantly forgets that the tongue is thrust out of his mouth, and the trains of the usually catenated muscular motions being for the time interrupted, he feels no impulse to replace it in its natural situation."

"There seems here abundant ground for the belief, that the first impression of the typhus poison, or exhalation, when applied to our bodies, is always made on the nervous fibre, or tissue, and that hence arise those various derangements of its grand centre, the cranial brain, sometimes the spinal, that form such prominent features of the whole disease. I shall begin with headach. This is a symptom almost invariably accompanying the commencement of typhus, yet I do not know that it furnishes a basis for prognostic. We observe it of different degrees of constancy and intenseness, yet generally yielding to remedies long before the termination of the fever. If peculiarly violent, it may indicate some serious disorder of the brain, or its membranes, and so far, must be considered of evil import; yet we seldom can draw from it any certain prediction concerning the final issue. Delirium, its frequent concomitant, or sequela, is a far more important, or terrifying, appearance. This, too, admits of great variety, in point of severity, and continuance. Sometimes it appears only during night, and ceases during day; at other times, it harasses the patient equally during both periods. At one time it is violent and furious, requiring coercion, and the strait-jacket; at another, quite mild and tractable. The hallucinations of mind attending it are no less variable. Sometimes we observe the trains of ideas to be sad and mournful, at other times cheerful, joyous, and full of exultation. Patients occasionally think they are pursuing their ordinary trades and occupations, with all their usual cares, and anxieties.* One man appeared actuated by deep remorse and despair, insisted that he was condemned to be hanged, and was constantly talking about the day of execution. In some, the predominant feeling is terror, and terror in the most intense degree; when you approach their bed, they scream out, start up with the most agonizing fear pictured on their countenances, try to escape, or hide themselves beneath the bedclothes.†

"Under all these, or other shapes, delirium must ever be regarded as an alarming symptom. How far one variety may indicate greater danger than another, I confess myself unable to decide. Judging

* "Fever hospitals abound in such instances. One of Dr. Cheyne's patients in the Hardwicke Hospital, Dublin, was a cooper. He was caught one night taking asunder the frame of his bed, in order to make a tub out of the spars.—A cowherd, confined in the Cork Hospital, mistook the sick around him for so many cows, and was frequently trying to rouse them by means of his accustomed cries. (Dr. Pickells, in Irish Med. Trans., vol. iii. p. 108.)—A carter in our own wards was incessantly driving his cart, and talking to his horse. This hallucination continued no less than four days and nights; but the man recovered.

† "A patient in our female ward, after escaping from fever, was seized with acute parotitis, which occasioned violent delirium. Under its delusion, she insisted on having her head cut off; not with any view of self-destruction, for that never entered her mind, but solely, it appeared, for relief of the pain. To obtain her purpose, she first of all applied to the clerk; but, being unsuccessful, had recourse to me at the usual visit. Being again refused, she burst into tears, exclaiming that she was shamefully and cruelly used, and that nothing was done for fever patients in the Glasgow Infirmary."

from my own experience, where extreme and unappeasable terror is the predominant feeling, I would consider it of fatal augury. Under this unfortunate hallucination, I never knew a single instance of recovery. At the same time, the examples that have fallen under my notice are too few to draw from them any certain or general conclusion.*

"Equally to be dreaded with active delirium, is that profound coma, where the patient cannot be roused—where the pupils are either morbidly contracted, or dilated, and where the pulse is, at the same time, tardy or intermittent. No man will view these symptoms without the most serious apprehension. Luckily, they do not occur with very great frequency in typhus. In opposition to all this, however, there is a species of coma, that, however paradoxical it may appear, I hold rather desirable than to be dreaded. The patient here lies in a state of mild stupor, or, more correctly, seems buried in a profound and imperturbable sleep. The senses, for the time, may be said to enjoy a holiday; for he neither tastes, nor smells, nor feels, nor sees, nor hears. What passes within we know not, but there is complete interruption to every disturbance from without. All external impressions pass over him unheeded and unfelt. When you rouse him up, and this capability of being roused forms the distinction between this and the other dangerous coma above noticed, he mutters a few words, and then sinks back into his wonted repose. This singular state, the *coma somnolentum* of nosologists, will often continue for several days, and patients under it seem for a time free from all contest with the fever, and as it were to lay in a reserve of strength to meet the future contingencies of the disease; at any rate, it is rather a favorable than unfavorable sign. I never knew an individual die who had passed fairly through its ordeal.

"In direct contrast or contradistinction to this *coma somnolentum*, we sometimes meet with another affection, for which, however, nosology has not yet furnished a name, where the different senses, instead of being preternaturally obtuse and torpid, are found, on the contrary, morbidly and inordinately acute. Common impressions, in place of being indifferent or pleasurable, excite nothing but uneasy feelings. The mildest light is painful to the eye—the softest sound is torture to the ear. But the chief seat of the evil seems to lie in the nerves of the skin. So immoderately tender and sensitive are these nerves, that the patient cannot suffer himself, even for a moment, to be touched. If you lay hold of his hand in

* "Upon the whole, there is no single symptom of fever that indicates greater danger than delirium: every experienced practitioner, I believe, will subscribe to this opinion. Dr. Grattan, of Dublin, asserts that if this evil subsist so long as three days and three nights, there are few persons that will escape with life, such is the exhaustion its constant excitement produces. (Irish Med. Trans., vol. iii. pp. 440-41.) This perhaps is taking too gloomy a view. You have seen various cases treated successfully where the symptom was of at least three days' duration."

order to feel his pulse, he instantly screams out, and entreats you, sometimes with anger, sometimes almost with tears, to desist. The same unnatural sensitiveness extends over the whole body. The pressure of the bed or bedclothes is intolerable, and he is constantly changing his position, which, instead of alleviating, only enhances his uneasiness. Jactitation is extreme, and he is entirely deprived of sleep. It is needless to remark how much such a condition must necessarily aggravate every existing symptom of fever. As to the exact prognostic inference, or how much danger it may indicate, I have seen too few instances of this curious affection to determine. Experience, though limited, has taught me to regard it of dangerous, or fatal tendency. Two cases have appeared in our wards during the last twelvemonth, both corroborative of this conclusion; one, that of a man where, though it assumed a chronic shape, the issue was finally unfortunate: the other that of a female, where it was highly violent and acute, and where the patient died the very next day after its appearance.

"In the two singular nervous derangements above described, we found the whole of the external senses, without exception, perverted: occasionally, again, it is only two of them—the eye and the ear—that suffer. Deafness is a most common symptom, nor do I think it bodes danger: some practitioners, on the contrary, regard it as favorable. It will at least exempt the patient from the irritation of noises. It is often exceedingly inconvenient to the practitioner, however, by preventing due inquiries into his patient's condition. As for the eye, the usual perversion of vision consists in spectra of small size floating about in the atmosphere around, more particularly assuming the appearance of flies (muscæ volitantes), and which, we are told, the sick are perpetually endeavouring to catch. Of this symptom I know but little from personal experience; I have no recollection about it, nor do I think I have seen it above four or five times in my life.

"Considering with what a deadly grasp typhus poison lays hold of the brain and nerves, we need not wonder that convulsive and paralytic affections should appear so frequently in the disease, or should constitute some of its principal symptoms. Among the first may be chiefly noted those short and jerky motions of the forearm and hands so familiar to the malady, which, as they occur in a place where there are so many tendons, both long and short, are usually designated by the sort of technical phrase, *subsultus tendinum*, or jumping of the tendons. Similar convulsive twitches are observable in other parts, as in the extremities, and more particularly about the muscles of the upper lip, where they are by no means unfrequent. As to the prognosis, authors generally take a gloomy view, considering them as of very dangerous import: a conclusion, however, not warranted by experience. In our ordinary typhus they are seldom found altogether wanting; and, so far as my own observation reaches, as many patients recover after them as of those who never experienced them at all. Along with subsultus tendinum, is also commonly arranged another sign wi.h like technicality, termed *carpologia*, or chaff-gathering, because it consists of a similar series of brief shaking motions, as if the sick were picking fragments of straw, or chaff, from the bedclothes. Perhaps this affection has been wrong placed, and should be considered a mere visual hallucination, rather than a convulsion. Whatever be its nature, it is of rare occurrence. I have certainly met with it, but by no means frequently.

"As to the paralytic affections induced by the poison of typhus, none is of more deadly omen than palsy of the sphincter muscles of the bladder and rectum, giving rise to an unconscious and involuntary discharge, in bed, of the urine and fæces. The total oblivion here of all ordinary or established feelings or habits, shows the deep injury that has been inflicted on the sensorial organs, and the risk to life is in proportion. I know of no symptom that ought to inspire a greater despair of recovery in the mind of the practitioner. Exert ourselves as we may, it will generally be found an almost certain precursor of destruction. Another paralytic affection occurs in the bladder itself, though apparently attacking a different portion of the organ. In the former instance, the sphincter alone was in fault, but here it is sound, while the paralysis affects the muscular fibres of the interior, so as completely to disable the viscus from expelling its contents. There is thus produced what may be called a typhus dysury; and, if we believe high authority, it is of more evil augury than even palsy of the sphincter. The late Dr. Gregory, of Edinburgh, used to declare, in his lectures, that he never knew a single patient survive after the occurrence of this symptom.* The disease here I have imputed to the bladder alone, without implicating the kidney, and I think on good grounds. The urine which we are often obliged to draw off by the catheter, affords sufficient testimony that the gland itself has been performing its function.

"Another, and most dangerous paralytic affection in fever. I had almost forgotten to mention, namely, palsy of the muscles of deglutition. We have lost four or five patients mainly from this cause. The power of swallowing being lost, all access of wine or other medicine to the stomach, of course, ceases. Enemata afford but an imperfect resource, and I commonly found the sick too weak and exhausted for Jukes' apparatus.

"Among other effects of typhus poison, none is more striking than the extreme prostration of strength it is apt to occasion, reducing the whole frame as it were to a state of almost infantine imbecility. This happens sometimes at the beginning, more frequently during the currency of the disease, either from the more gradual operation of the virus, or from simple exhaustion. When it exists in excess, and then it is to be highly dreaded, it becomes

* "There is no rule however without exception, particularly in medicine. We have met with two cases, where, though the disease was far advanced, yet the patient survived this dangerous symptom: one was that of Mary Murray, (vol. ii. p. 89.) The name of the other has escaped my memory."

easily cognizable by the posture of the sick. If you find your patient lying supine, or stretched flat upon his back, for days together, and unable to stir a muscle, there can then no doubt remain that this perilous, and distressing symptom has fairly taken place. You must endeavour by every mean in your power, to support the languishing strength, though too often your efforts will be vain. The fatal debility continues to creep on, from day to day, or from hour to hour, encroaching more and more on the powers of life, till at last it becomes a mere step or prelude to dissolution." (P. 19.)

(To be continued.)

---

*On the Practical Prevention of Dry Rot in Timber; being the Substance of a Lecture delivered by* Professor FARADAY, F.R.S. &c., *at the Royal Institution, February* 23, 1833.—8vo. pp. 24.

THE subject treated of in this Lecture is one of vast importance; one to which persons have hitherto turned their attention with but little practical advantage; and, although numerous experiments have been tried, to prevent or retard the destruction of timber by what is termed *dry rot*, the pest of the British navy, none of the plans proposed have been successful.

Such being the state of the case, we confess that it was with some misgivings that we entered on an examination of the evidences brought forward by Mr. Kyan in favour of a process, by which he promises to prevent dry rot occurring in sound timber, and even to arrest its progress in that which is in part decayed.

We did not, however, come to this subject unprepared to enter fully into the details; for some years ago we ourselves made many experiments upon different kinds of timber, in order to ascertain the causes of their relative durability and decay.

The results of these experiments were published in the Journal of the Royal Institution, and the conclusions then arrived at have perhaps led us more fully to appreciate Mr. Kyan's process, than, without such preliminary knowledge, we should have been capable of doing.

We then shewed that, although wood of all kinds is formed of tubes and cells, that the cavities of these are more or less completely filled in the timber afforded by different trees, giving to it the well-known different degrees of weight and firmness. That upon the nature of the matters stored up in these cells the other physical qualities of the wood, as its colour, smell, and taste, depend. Dutrochet shewed that the *woody* structure of ebony is as white and as light as poplar, and that its weight and black hue is owing to the dense matter contained in the cellular structure, which may be dissolved and removed by proper solvents, leaving the ligneous structure entire. From these and other observations the philosophy of decay can be easily understood; for, if immature or ill-seasoned wood be employed, the contents of the cells will undergo various chemical changes, such as putrefaction, &c., forming a fit and favourable soil for fungi, not only for the true dry-rot (Merulius lacrymans), but for a whole host of other wood-destroying plants.

In the essay already referred to it was also shewn that the reason why certain woods, which are durable in dry situations, are extremely perishable in wet, is that the materials laid up in the cells, and on which the firmness of the wood depends, is in some easily, and in others sparingly, or not at all, soluble in cold water. Thus this matter is much less soluble in good oak than in deal or beech: and also in some kinds of oak than in others. For example, our British ship-building oak (*Quercus pedunculata,*) forms in its ligneous cells matter scarcely at all soluble in water; whereas another oak, also growing in this country, but much more abundant on the continent of Europe, forms matter easily soluble. Hence the timber of the one will endure unchanged for ages; while the other, if exposed to the vicissitudes of the weather, heat and cold, wet and dry, will decay with rapidity. To the introduction of this inferior oak, *i. e.* to the consumption of so much more foreign timber than usual in our dockyards, may be traced the comparative perishability of many modern-built ships: for vessels, instead of lasting, as they have been formerly known to do, for half a century, are now often broken up within two, three, or five years; and some have been obliged to undergo a thorough repair even before they were launched.

Now, from what has been said, it is evident that if the matter which is soluble in perishable woods, or that which is prone to run into decomposition in immature timber, could be rendered in the one case insoluble, and in the other the tendency to putrefaction be restrained, both would be brought into a condition similar to that of wood which is naturally of an enduring kind. And this desirable object appears to have been attained by Mr. Kyan, who has our warmest thanks for the energy with which he has performed the investigation, as well as for the benefits which his success must confer upon society.

Having rendered, as we hope, the rationale of decay intelligible to all, we shall now proceed to give some extracts from

Professor Faraday's lecture upon Mr. Kyan's process, which, we feared, from the concise manner in which the subject was obliged to be treated, might not be clearly understood without some such prefatory remarks.

After a few introductory sentences, Dr. Faraday observed,

"With regard to decay in general, and to that of wood in particular, which gives rise to the destruction now to be guarded against, it was hardly possible to say anything definite as to the cause of that decay, for it seemed to him that cause and effect almost replaced each other; that is, what was a consequence at one time, was at other times a cause of the final ruin of those fibrous matters which are used for domestic purposes. On reference to the different specimens around him, instances would be seen of rapid and extensive decay, which it is the object of the process he had to bring before them to prevent. For instance (said Pr. F.), here is exhibited a beam of wood in which the one part is rapidly passing to decay, and the other part is sound, or nearly so. There is a piece of wood on the table made into a thin plank, and laid over the internal woodwork. It has been painted outside, and appeared to the eye to be good, but after a short time the rot caused it to decay: the rot had run from the interior to within the twentieth part of an inch of the paint, which, aided by the air and light, had stopped its progress. Pr. Faraday shewed another specimen which had been sent to him by Professor Burnett, of the results of the same kind of decay, or what is called the Dry Rot fungus. It was the most magnificent he had ever seen, and was formed in a sort of gutter or wooden trough across a large room, the conservatory of his Grace the Duke of Norfolk. The fungus had prospered so much, that even its fructification was fully developed. Those who might wish to examine it closely would see that the plant was extending itself from place to place, and producing here and there aggregations of seed-vessels consistent with its organic arrangements. Another specimen, was a case of decay from animal operations, which had been going on at Woolwich; it was taken from a large mass of timber, so penetrated by the worm that it appeared to be like a piece of sponge. There was another piece similarly circumstanced, which had been a part of one of the timbers belonging to Brighton Pier. It in the first instance had been fifteen inches square before being placed under water; and it was wonderful to observe how, by decay and the action of various insects, it had been brought to its present state. Another specimen, was part of a mast of a vessel, which, though to appearance sound on the outside, was in the inside gone, as if it had been hollowed out by a workman's tool: the decay had taken away the strength of the mast. These being the conditions to which timber had been brought in domestic and naval applications, it has been admitted in all times a desideratum to obtain some substance which should prevent such kind of action." (P. 4.)

After adverting to the various other plans which have been proposed for the prevention of dry rot, he continued by saying,

"He might dispose of all these, because, having been tried fairly and having failed, it was presumed the trials had not led to any final application. He should take up only that one which he had considered, (as far as its chemical principles were concerned, and as far as it was connected with the case,) and thought to be borne out by the previous knowledge of the substance used, as being sufficient for the purpose proposed; requiring only to be tried to justify the full assurance of its use. The process he had to lay before them depended upon the application of an anti-destructive, which had been long known as a very active body in all such cases, namely, Corrosive Sublimate; for every anatomist and every visitor of an anatomical museum knows that corrosive sublimate was, and is, used from time to time to prevent the decay of the most delicate organic tissues and parts, even such things as the brain itself, which are liable to putrescence; and by the application of this metallic preparation they can be prevented from going to decay, and be preserved for any length of time. This was a matter of common knowledge; and Sir Humphry Davy and other chemists have referred to the power of corrosive sublimate, and the nature of its application. An application was made to him (Pr. F.) many years ago, which afforded him great gratification at the time, for the purpose of attempting to preserve Earl Spencer's library from decay by the book-worm. It had been proposed to apply corrosive sublimate; and Sir Humphry Davy was referred to, who honoured him by asking his opinion whether the volatility of that body, (called corrosive sublimate from the very circumstance of its being purified by volatilization,) would not be liable to create such an atmosphere where the books were placed as would be injurious; and which, though not felt for one, two, or three days, would affect those who wrote and studied in the place. His opinion then was against the corrosive sublimate, which agreed with that of Sir H. Davy. They both thought it might possibly be hurtful to those beings who were more worthy of attention than the maggots in the books.

"A gentleman of the name of Kyan, considering the property of corrosive sublimate, proposed to apply it to timber for the prevention of the dry rot: that is, cases of decay, whether they arise from the action of the seeds of cryptogamous plants, vegetating in the wood, or from the presence of the albuminous parts of the tree. Mr. Kyan thought the evil might be stopped; that the commencement even might be prevented by the application of corrosive sublimate, in consequence of the chemical combination which takes place between the corrosive sublimate and those albuminous particles which Berzelius, and others of the highest authority, consider to exist in and form the essence of wood: which being the first parts that run to decay, cause others to decay with them. Mr. Kyan's conviction was such, that he went to the Admiralty to place it before them. They required certain trials to prove the soundness of the application, which trials he (Pr. F.) would now have to bring forward. After these were carried on for two or three years, the Admiralty advised Mr. Kyan to take out a patent; and were still engaged in watching the progress of these trials since that period.

"He would now tell them how it was proposed to prepare timber, and what the results were. The proposition was, to soak the timber itself in a solution of corrosive sublimate. Pr. F. then shewed the model of what is termed a tank, in which the timber is to be immersed in the solution. He said the meeting must not be struck with the name of that which a few years ago was rather expensive, but now a cheap application, for a pound of it did not cost much in proportion to the good that would ensue; which he (Pr. F.) thought would be fully confirmed by the result, after a few years' experience. A solution of this substance is made, and timber is placed in the vessel. The timber is held down in such a way, that, when immersed, on the fluid being pumped in, it cannot rise, but is kept under the surface, there being beams to retain it in its place. There it is left for a week, after which the liquor is pumped off, the wood is removed, and it comes out in the state of the samples before him. This being done, the timber is dried, and said to be prepared: it has been applied by Sir Robert Smirke to the new buildings in the Temple, and has been tested in a very extraordinary way, of which some account will now be given.

"Besides the application of corrosive sublimate to timber, it has been applied to various fabrics not composed of wood, as, for instance, canvass, cottons, tows, and hemp, to prevent their decay. Before him were some of the pieces submitted to trial by order of the Admiralty, three years ago, in the fungus-pit at Woolwich, which he (Pr. F.) went the other day to see opened. It was a pit dug in the yard, and enclosed by wood on all sides, having a double wooden cover; it was damp of itself, and into this were put various kinds of wood of which they wished to make trial. One specimen was a piece of timber which came out at the end of three years as sound as it went in; but the unprepared timber had decayed up to the very joint. No part of it had been left. It had decayed and become rotten throughout, but the piece before them was left whole and sound, and fit for the construction of vessels. Last week he saw a large cube of wood which had been there first for three years; it was taken out, examined, and put in for two years more, altogether making five years. That cube of wood was again taken out, and examined by him on Tuesday (the 19th February); it was perfectly hard and sound. There was no sign of decay in that wood which had been submitted to the rotting action for five years, nor of that destruction which seems to have come on so soon in the same pit with other pieces of wood.

"Sir Robert Smirke had a couple of posts put up under a dropping eave, and both were exposed to the same actions. After a certain time one of them decayed; the other still stands, having been preserved by the power of this substance. There were before him some specimens of canvass and cotton which had been in various ways exposed to damp, placed in a cellar on the 10th December, 1832, and left till the 21st February, 1833: they were taken out for the purpose of being exhibited that evening. Another, a prepared and unprepared piece, which had been coiled up in a cellar from the 15th December, and left to the 21st February, 1833. The opposite effects seen were produced by the same circumstances of exposure on prepared and unprepared calico. One was as it went in, but the other was the calico corresponding to it, which had rotted and decayed. It was not possible to unfold without destroying it, yet it had been similarly exposed as the first. Nothing could here deceive regarding the appearance of mildew; the difference between the two was so evident, that no person could make a mistake about them: one had run into decay and was falling to pieces.

"Now he (Pr. F.) must confess, for his own part, that he was perfectly satisfied of the preservative effects of corrosive sublimate. He knew beforehand that it would preserve things far more liable to decay than timber, canvass, and cotton, but had had doubts as to its application. His query, in the first place, had been, was this preventive or anti-corruptive substance of such a nature that it could remain there, or was the effect not merely a temporary one, that would pass away after a time; and in the timber of vessels which were exposed to the bilgewater, and other water, where vessels were not coppered, was the sublimate not likely to be removed, and its effects destroyed? And, if that were not the case, was there not a fatal injury that might arise from the production of a noxious atmosphere? The answer was 'No, we expect not; for chemical combination has taken place between the corrosive sublimate and the body to be preserved; and it prevents the destructive power going on, by combining and forming a new chemical compound with the albuminous matter of the wood.' This being to him at that time a doubtful opinion, he wished to obtain some proofs for his own satisfaction." (P. 7.)

The lecturer then proceeded to detail experiments which he had made, and which had convinced him of the truth of the anticipation above expressed. He proved that the corrosive sublimate enters into chemical combination with the destructible matter of the wood, converting it into insoluble and enduring timber.

"Therefore it was not to be expected, when they thus come together by a kind of chemical union, that the properties of the original substance should be free to act. No; part of the properties of the corrosive sublimate were subdued, and, in fact, the substance was not in that state in which it could be volatilized, or removed at ordinary temperatures. Very simple proofs would be satisfactory. It occurred to him to take some of the prepared canvass, washing it thoroughly in water several times, to see if the sublimate could be removed from the cloth. Calico was taken in preference, because it seemed more easily to allow the removal of the new chemical combination, if that could be effected. He thought it would be the most conclusive proof to be obtained; for, if the properties of the compound would resist the application of water in calico, they would of course do so in timber, where the substance, combining with the sublimate, was contained in the pores of the sap-vessels. Not being satisfied with the work of others, he washed the prepared calico himself in water: he then shewed a board on which he had put this washed calico; on one part was shown the portion of prepared calico which had been washed, and on the other a similar piece unprepared. The latter was covered with a coat of fungus nearly half an inch thick, the former was quite free from it. To be certain whether the calico so prepared and washed did retain any mercury, and to assure himself that it had not been accidentally so placed in the damp cellar that it was left uncorroded, while the unprepared piece was corroded, a portion was treated with dilute nitric acid, by which means he could, though to the injury of the whole material, separate the mercury. By washing the prepared calico in water, he was unable to obtain any portion of the metal, but the mercury was separated by nitric acid, showing that it had been in combination: and there was no reason, after such trials, to suppose it could be washed away, or rise as vapour, and be injurious. These two or three experiments influenced him in giving his judgment, that the process would be found effectual in preserving timber. He added, I think the improvement so great as fully to justify its extensive application." (P. 12.)

